# The Potential of *Siraitia grosvenorii* to Promote Bone Regeneration via Modulating Macrophage Polarization: A Network Pharmacology and Experimental Study

**DOI:** 10.3390/ijms26083609

**Published:** 2025-04-11

**Authors:** Yuying Mai, Linhui Huang, Yang Qiao, Yuan Qin, Lufei Wang, Hongbing Liao

**Affiliations:** 1 College of Stomatology, Guangxi Medical University, Shuangyong Road 22, Nanning 530021, China; maiyuying@stu.gxmu.edu.cn (Y.M.); 202010151@sr.gxmu.edu.cn (L.H.); qiaoyanggg@outlook.com (Y.Q.); qinyuan777@126.com (Y.Q.); 2 Guangxi Key Laboratory of Oral and Maxillofacial Rehabilitation and Reconstruction, Nanning 530021, China

**Keywords:** *Siraitia grosvenorii*, bone defect healing, network pharmacology, macrophage polarization, osteogenic differentiation

## Abstract

*Siraitia grosvenorii* (SG), a traditional Chinese medicinal herb, possesses immunomodulatory and osteoinductive properties, yet its pharmacological mechanisms in bone defect repair remain largely unexplored. This study investigates the therapeutic potential of SG through a combination of network pharmacology and experimental approaches. Active compounds were identified using the Traditional Chinese Medicine Systems Pharmacology (TCMSP) Platform, and protein interaction targets were predicted. Molecular docking and dynamics simulations assessed interactions between SG compounds and critical targets. In vitro, RAW 264.7 macrophages treated with SG-conditioned medium exhibited enhanced M2 polarization and reduced inflammation, promoting osteogenic differentiation of co-cultured MC3T3-E1 cells as evidenced by increased alkaline phosphatase activity. In vivo, scaffolds loaded with low-dose or high-dose SG (LSG/HSG) significantly improved bone regeneration in rat calvarial defects, with HSG achieving near-complete repair and mature trabeculae at 8 weeks, alongside decreased CD86 and TNF-α levels and increased IL-10 expression. Network pharmacology identified 33 shared targets related to bone regeneration and macrophage polarization, with kaempferol and beta-sitosterol demonstrating strong binding affinities to targets such as TNF, PTGS2, and CASP3. These findings highlight the potential of SG in enhancing bone defect repair and its implications for regenerative medicine.

## 1. Introduction

Bone defects continue to challenge modern medicine, particularly in cases of extensive injury where impaired healing trajectories often result in chronic pain and functional disability, according to epidemiological studies [1,2]. Advances in tissue engineering, particularly through the application of biological scaffolds, have shown promise in promoting bone regeneration; however, challenges such as prolonged degradation rates, inadequate osteogenic potential, and insufficient vascularization of these scaffolds remain [3,4]. Intriguingly, the mechanisms underlying bone defect repair are multifaceted, with recent research highlighting the pivotal role of macrophage polarization, which is linked to governing inflammation, tissue remodeling, and regenerative processes [5,6]. Despite ongoing investigations, the precise mechanisms driving macrophage polarization in bone repair are poorly understood. Emerging evidence suggests that Traditional Chinese Medicine (TCM) may modulate macrophage polarization, presenting a promising therapeutic avenue that warrants further exploration [7,8]. Notably, controllable phase transition gelatin-based (CPTG) bioadhesives enhance adhesion energy and immune modulation, while hydrogel microneedle systems (6-G@MN) promote angiogenesis and collagen deposition while rebalancing macrophage populations [9,10]. These innovative approaches highlight the potential of integrating TCM with modern biomaterials, ultimately contributing to developing effective strategies for enhancing bone defect healing and improving clinical outcomes.

*Siraitia grosvenorii* (SG), a traditional Chinese medicinal herb, is renowned for its therapeutic properties, particularly its immunomodulatory effects that enhance inflammation resolution and healing processes [11]. The extract modulates immune responses by regulating key cytokines, such as tumor necrosis factor-alpha (TNF-α), interleukin-1 beta (IL-1β), and interferon-gamma (IFN-γ), while promoting anti-inflammatory markers like CD206. Furthermore, SG inhibits the mitogen-activated protein kinase (MAPK)-nuclear factor-kappa B (NF-κB) signaling pathway, which is crucial for mitigating excessive inflammation and fostering a tissue repair-conducive environment [12,13,14]. Mogroside V, the bioactive compounds of SG, regulates insulin resistance via the phosphoinositide 3-kinase (PI3K)/Akt pathway, while flavonoids such as kaempferol and quercetin exhibit substantial antioxidant activity [15]. Recent findings suggest that SG reduces pro-inflammatory cytokine production and upregulates tissue repair factors in osteoarthritis, indicating a potential role in macrophage polarization [16]. Nevertheless, the role of SG in bone regeneration remains largely unexplored, highlighting a significant gap in current literature and underscoring the need for further investigation into its mechanisms and therapeutic applications.

Cyberpharmacology, which employs systems biology and bioinformatics, encompasses a broad range of applications aimed at elucidating the mechanisms underlying drug efficacy and the discovery of novel therapeutics [17]. Techniques such as molecular docking elucidate interactions between drug molecules and receptors, predicting binding affinities [18]. Molecular dynamics (MD) simulations are employed to evaluate the stability and adaptability of ligand–receptor complexes [19]. This study seeks to elucidate the mechanisms by which SG promotes bone repair through the integration of network pharmacology, molecular docking, and MD simulations, thus offering a novel perspective for clinical therapeutic strategies.

## 2. Results

### 2.1. Identification of Bioactive Compounds in SG

A search of the Traditional Chinese Medicine Systems Pharmacology (TCMSP) database identified 182 active ingredients in SG. From these, 11 compounds were selected based on specific criteria, including an oral bioavailability (OB) of ≥30% and a drug-likeness (DL) score of ≥0.18 (Table 1).

### 2.2. Identification of Gene Targets for SG

**Figure 1:**
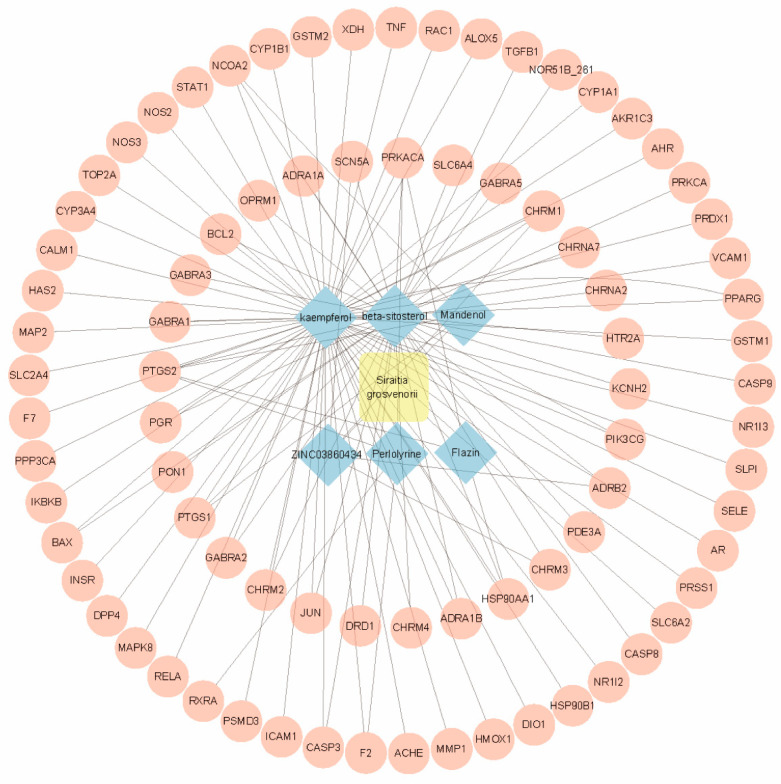
The ‘Herb–Chemical composition–Target’ network. A total of 6 active compounds have been screened in SG, corresponding to 113 bioactive targets.

The TCMSP platform was utilized to identify gene targets associated with the active ingredients previously mentioned. After eliminating duplicates, a total of 113 gene targets for SG were identified. These data were subsequently analyzed using Cytoscape software(version 3.10.2), resulting in the construction of a network comprising 89 nodes and 118 edges; the nodes represent the gene targets related to the active ingredients, while the edges signify the interactions or relationships between these gene targets (Figure 1). Notably, the six active ingredients with the highest degree of interaction were MOL000422 (kaempferol), MOL000358 (beta-sitosterol), MOL001494 (Mandenol), MOL001749 (ZINC03860434), MOL002140 (Perlolyrine), and MOL009295 (Flazin), corresponding to 60, 38, 3, 4, 4, and 3 gene targets, respectively. These findings suggest that the various active ingredients within this network interact with multiple targets, highlighting the multi-component and multi-target nature of SG pharmacological effects.

### 2.3. Identification of Therapeutic Targets for SG in Bone Regeneration and Macrophage Polarization

**Figure 2:**
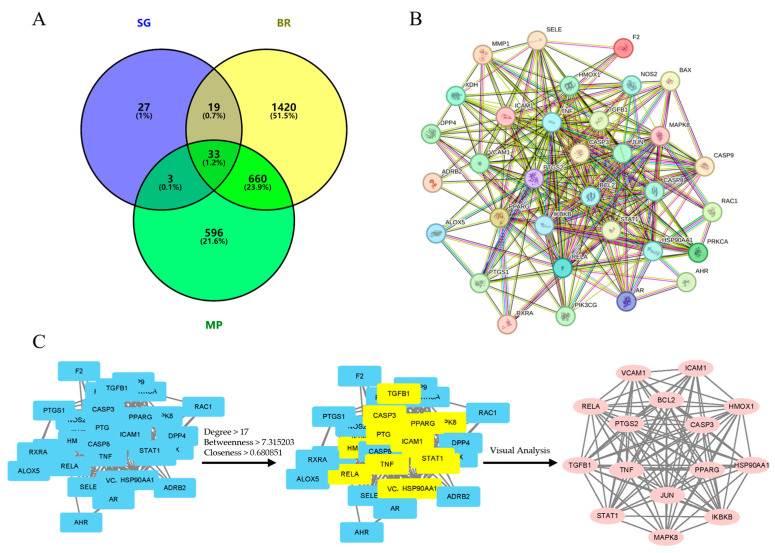
Venn plot, PPI network diagram, and screening topology of core targets of SG treatment for bone defects. (**A**) The Venn diagram illustrates the crosstargets of SG in bone regeneration and macrophage polarization. (**B**) The PPI network displays the interactions among the 33 crosstargets. (**C**) The topological representation highlights the core targets involved in SG treatment for bone defects.

To identify therapeutic targets associated with bone regeneration (BR) and macrophage polarization (MP), we conducted a targeted search in relevant databases. The UniProt database was utilized to standardize the nomenclature of gene targets. This process yielded 2132 targets related to BR and 1292 targets concerning MP from the GeneCards database. It was found that there was a match between these targets and 111 SG targets. These targets were then compared with 111 targets from SG, resulting in the identification and mapping of 33 crosstargets. The Venny 2.1.0 platform was utilized for the identification and mapping of these crosstargets (Figure 2A).

### 2.4. PPI Analysis

The 33 crosstargets were imported into the STRING database for analysis, leading to the construction of a protein–protein interaction (PPI) network consisting of 33 nodes and 264 edges, generated using Cytoscape software (version 3.10.2). In this network, nodes represent target proteins, while the connecting lines indicate their interactions (Figure 2B). The PPI network emphasizes core targets with the highest interaction values, defined by the following criteria: Degree > 17, Betweenness > 7.315203, and Closeness > 0.680851 (Figure 2C). These criteria have identified the top protein targets that play a central role in the network, and those proteins are as follows: TNF, PTGS2, PPARG, CASP3, JUN, HSP90AA1, BCL2, RELA, TGFB1, ICA1, STAT1, HMOS1, VCAM1, IKBKB, and MAPL8 (Table 2).

### 2.5. Gene Ontology (GO) Enrichment Analysis

**Figure 3:**
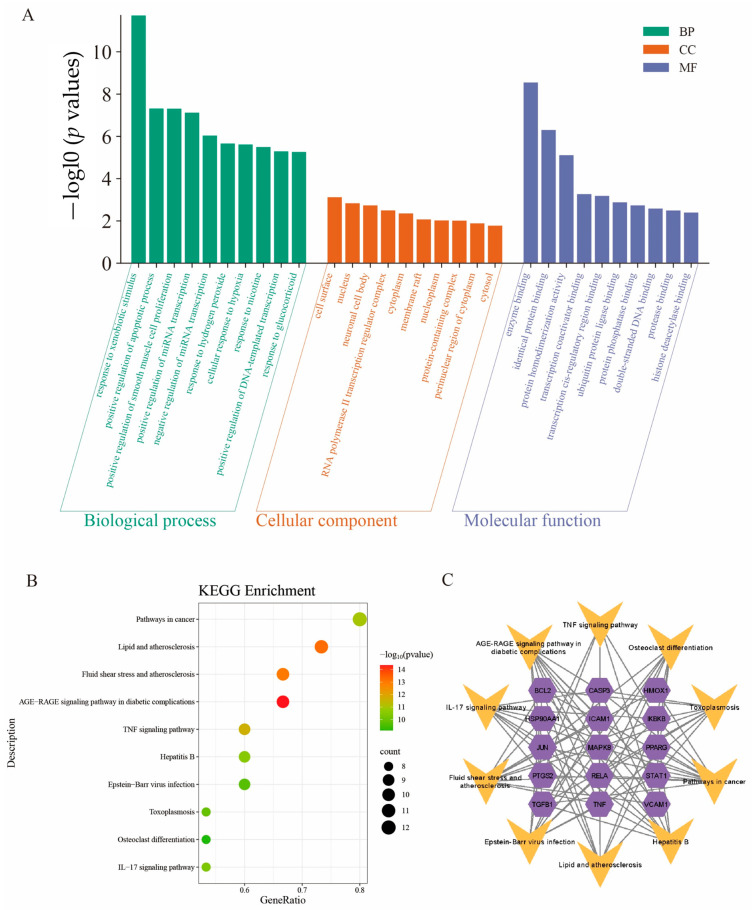
Gene Ontology (GO) and Kyoto Encyclopedia of Genes and Genomes (KEGG) pathway enrichment analyses. (**A**) GO enrichment analysis, including biological processes, cellular components, and molecular functions. (**B**) KEGG pathway enrichment analysis. The top 10 entries that take —logl0 (*p* values) are shown by the bubble chart. (**C**) “Target gene–KEGG pathways” network. Genes are represented by purple hexagons and pathways by yellow V shapes.

The core targets were subjected to Gene Ontology (GO) enrichment analysis, leading to the selection of the top 10 GO-enriched categories based on their *p* values, which were subsequently illustrated in a two-dimensional histogram (Figure 3A). A total of 187 enrichment results were identified, comprising 144 biological processes (95.3%), 18 cellular components, and 25 molecular functions.

The ten most significant biological process (BP) terms revealed by the GO enrichment analysis included the following: response to xenobiotic stimulus, positive regulation of the apoptotic process, positive regulation of smooth muscle cell proliferation, positive regulation of miRNA transcription, negative regulation of miRNA transcription, response to hydrogen peroxide, cellular response to hypoxia, response to nicotine, positive regulation of DNA-templated transcription, and response to glucocorticoid.

The ten identified cellular component (CC) terms encompassed cell surface, nucleus, neuronal cell body, RNA polymerase II transcription regulator complex, cytoplasm, membrane raft, nucleoplasm, protein-containing complex, perinuclear region of cytoplasm, and cytosol.

The top 10 molecular function (MF) terms identified through GO enrichment analysis included enzyme binding, identical protein binding, protein homodimerization activity, transcription coactivator binding, transcription cis-regulatory region binding, ubiquitin protein ligase binding, protein phosphatase binding, double-stranded DNA binding, protease binding, and histone deacetylase binding.

### 2.6. Kyoto Encyclopedia of Genes and Genomes (KEGG) Pathway Enrichment Analysis

To further explore the possible functions of the targets, the KEGG pathway enrichment assessment was conducted. This assessment identified 15 pathways, from which the top 10 entries, based on *p* value, were selected to create a bubble map (Figure 3B). Subsequently, we constructed a “KEGG pathway–gene target” network (Figure 3C) to illustrate the relationships between the pathways and the corresponding targets.

### 2.7. Validation of Molecular Docking

**Figure 4:**
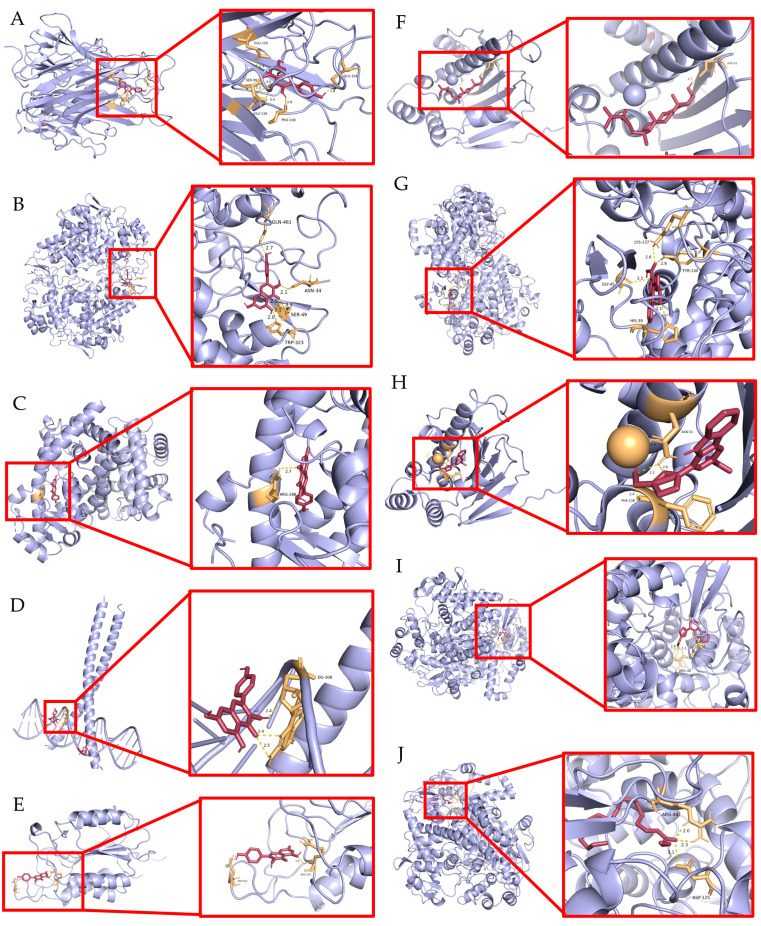
Molecular docking between representative components and core targets. (**A**) Molecular docking between TNF and kaempferol; (**B**) molecular docking between PTGS2 and kaempferol; (**C**) molecular docking between PPARG and kaempferol; (**D**) molecular docking between JUN and kaempferol; (**E**) molecular docking between CASP3 and kaempferol; (**F**) molecular docking between HSP90AA1 and beta-sitosterol; (**G**) molecular docking between PTGS2 and flazin; (**H**) molecular docking between HSP90AA1 and flazin; (**I**) molecular docking between PTGS2 and perlolyrine; (**J**) molecular docking between PTGS2 and mandenol.

The six core targets with the highest rankings (TNF, PTGS2, PPARA, JUN, HSP90AA1, and CASP3) were selected for molecular docking with five SG active ingredients. In this study, the molecular docking plots were generated using kaempferol, β-sitosterol, flazin, perillyrine, and mandenol. Lower binding energies are widely recognized as indicative of more stable interactions [20]. The molecular docking plots were generated using PyMOL software (version 2.5.7), and the results demonstrated that most active compounds displayed strong binding affinities to the designated nuclear targets (Figure 4; Table 3).

As shown in Table 3, kaempferol could bond to TNF, PTGS2, PPARG, JUN, HSP90AA1, and CASP3 with a Vina score of less than −7.00 kcal/mol. Beta-sitosterol could bond to PTGS2, JUN, HSP90AA1, and CASP3 with a Vina score of −9.20, −7.50, −6.60, and −7.30 kcal/mol, respectively. Flazin could bond to PTGS2 and HSP90AA1 with a Vina score of −9.60 and −8.20 kcal/mol. Perlolyrine and mandenol could bond to PTGS2 with a Vina score of −9.20 and −6.00 kcal/mol, respectively. However, it was observed that no hydrogen bond was formed between HSP90AA1 and kaempferol or between PTGS2, JUN, or CASP3 and β-sitosterol. Consequently, molecular docking plots from these components could not be generated. Thus, the components kaempferol, β-sitosterol, flazin, perlolyrine, and mandenol target TNF, PTGS2, PPARG, JUN, HSP90AA1, and CASP3 in SG, which may be the hub targets of SG components that promote bone formation.

### 2.8. MD Simulation Analysis

**Figure 5:**
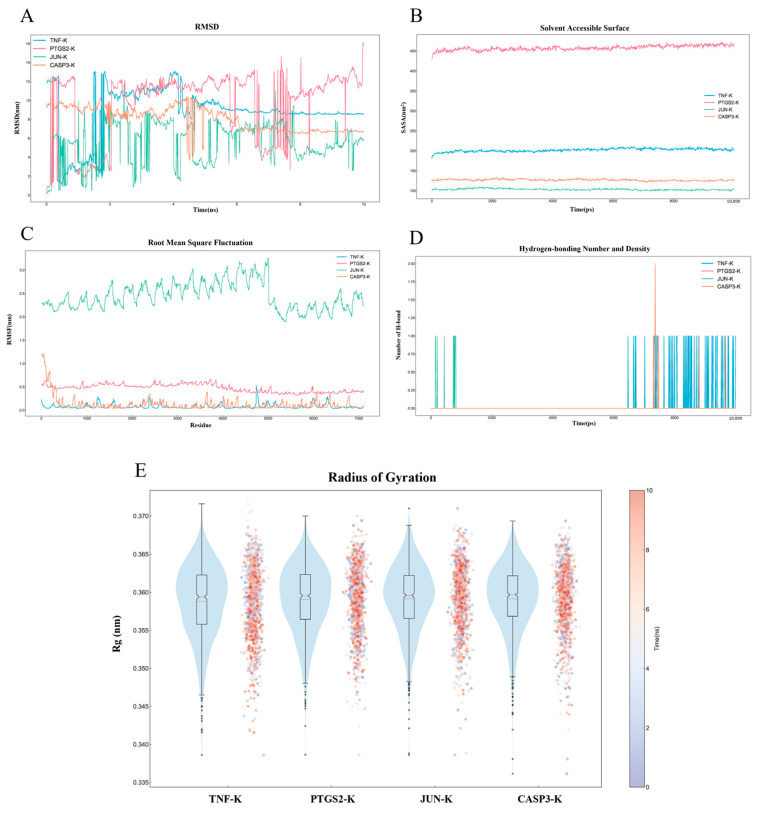
MD simulations of protein–ligand complexes. (**A**) Root mean square deviation (RMSD) curves of the complexes. (**B**) Solvent-accessible surface area (SASA) analysis. (**C**) Root mean square fluctuation (RMSF) curves of the complexes. (**D**) Hydrogen bonding number and density analysis of the complexes. (**E**) Radius of gyration (Rg) analysis.

To elucidate the stability and dynamic interactions of the protein–ligand complexes, four complexes with optimal binding activity were selected: kaempferol with TNF, kaempferol with PTGS2, kaempferol with JUN, and kaempferol with CASP3, which were subjected to MD simulations and visualized using DuIvyTools (Figure 5A–E). Root mean square deviation (RMSD) curves illustrate the conformational changes of the proteins during the simulations [21]. The average RMSD values were 8.778 ± 2.676 nm for the TNF-kaempferol complex, 9.675 ± 3.615 nm for the PTGS2-kaempferol complex, 5.606 ± 2.142 nm for the JUN–kaempferol complex, and 8.055 ± 1.274 nm for the CASP3–kaempferol complex (Figure 5A). The RMSD curves for the TNF–kaempferol and CASP3–kaempferol complexes stabilized after 5 ns, with fluctuation values of 11.89 nm, 16.03 nm, 11.57 nm, and 6.727 nm, respectively, indicating stable binding to the active sites without significant dissociation.

Solvent-accessible surface area (SASA) analysis of the protein trajectories showed that the solvent contact area remained stable for each complex, suggesting a strong binding affinity between the proteins and small molecules (Figure 5B). Root mean square fluctuation (RMSF) provides insight into the flexibility of individual protein residues [22]. The average RMSF values were 0.08097 nm, 0.4732 nm, 2.450 nm, and 0.1291 nm for the four complexes, respectively. The front regions of the proteins exhibited greater flexibility compared to other regions (Figure 5C).

The number and density of hydrogen bonds reflect binding strength, with average hydrogen bond counts of 0.05794, 0.000, 0.009990, and 0.01598 for the four complexes, respectively. The kaempferol–TNF complex demonstrated the highest density and strength of hydrogen bonds, followed by the kaempferol–CASP3, kaempferol–JUN, and kaempferol–PTGS2 complexes (Figure 5D). The radius of gyration (Rg) was analyzed to assess structural properties, providing information on mass, size, shape, and distribution. Rg values for kaempferol interacting with TNF, PTGS2, JUN, and CASP3 in their lowest energy conformations were 0.3588 ± 0.004784 nm, 0.3591 ± 0.004422 nm, 0.3592 ± 0.004416 nm, and 0.3591 ± 0.004345 nm, respectively. These values indicate stable molecular arrangements and minimal conformational changes upon binding (Figure 5E).

### 2.9. Cytotoxicity of SG

**Figure 6:**
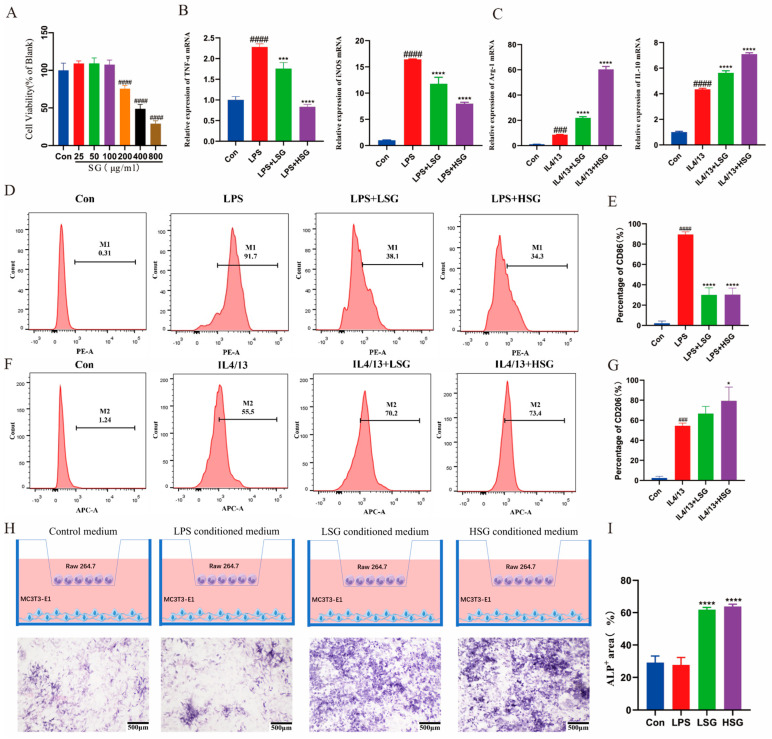
Effect of SG on macrophage polarization and osteogenic differentiation. (**A**) Effect of SG-conditioned media on RAW 264.7 macrophages. Cell viability was measured using the CCK-8 assay. (**B**) Quantification of pro-inflammatory cytokines in RAW264.7 cells following SG treatment, as measured by RT-qPCR. The expression levels of *TNF-α* and *iNOS* were assessed (n = 3). (**C**) Evaluation of anti-inflammatory cytokine expression in RAW264.7 cells. The mRNA levels of *Arg-1* and *IL-10* were analyzed using RT-qPCR (n = 3). (**D**–**G**) LSG/HSG conditioned media promoting the M2 polarization of macrophages. (**D**,**F**) Proportions of CD86 (M1 marker) and CD206 (M2 marker) detected by FCM after 48 h of treatment. (**E**) Quantification of the proportion of CD86-positive macrophages (n = 3). (**G**) Quantification of the proportion of CD206-positive macrophages (n = 3). (**H**) Schematic diagram of the co-culture system. ALP staining of MC3T3-E1 cells after 7 days of co-culture with different SG-conditioned media-treated macrophages. (**I**) The positive area in ALP staining (n = 3). Compared with the control group, ### *p* < 0.001, #### *p* < 0.0001. Compared with the positive control group, * *p* < 0.05, *** *p* < 0.001, **** *p* < 0.0001.

The impact of SG on the activity of RAW264.7 cells was assessed. As depicted in Figure 6A, exposure to SG at concentrations of 200 μg/mL, 400 μg/mL, and 800 μg/mL resulted in a significant decrease in cell viability (*p* < 0.01). For subsequent experiments, an SG concentration of 25 μg/mL was selected as the low-dose treatment group (LSG) and 100 μg/mL as the high-dose treatment group (HSG).

### 2.10. Effect of SG on the Inflammatory Factors in Macrophages

LPS treatment significantly elevated the expression of inflammatory factors TNF-α (*p* < 0.01) and iNOS (*p* < 0.05) in RAW 264.7 macrophages (Figure 6B). Conversely, IL-4 and IL-13 treatment resulted in increased expression of the anti-inflammatory factors Arg-1 (*p* < 0.01) and IL-10 (*p* < 0.05) (Figure 6C). SG treatment significantly reduced TNF-α (*p* < 0.01) and iNOS (*p* < 0.01) levels compared to the LPS group (Figure 6B). In addition, SG treatment markedly enhanced the expression of Arg-1 (*p* < 0.01) and IL-10 (*p* < 0.05) levels relative to the IL-4 and IL-13 group (Figure 6C). These findings indicate that SG effectively regulates the expression of inflammatory factors.

### 2.11. SG-Induced Macrophage M2-Type Differentiation In Vitro

Flow cytometry analysis (Figure 6F,G) demonstrated a significant increase in CD206 expression, a marker associated with the M2 phenotype, in the SG-treated group. Additionally, there was a reduction in CD86 expression, which is indicative of the M1 phenotype, in Raw 264.7 macrophages following SG treatment (Figure 6D,E). These findings confirm that SG effectively induced the differentiation of macrophages into the M2 type.

### 2.12. Osteogenic Differentiation of MC3T3-E1 Cells in a Co-Culture System

Alkaline phosphatase (ALP) staining results indicated that macrophages treated with SG-conditioned medium significantly enhanced the osteogenic differentiation of MC3T3-E1 cells (Figure 6H,I).

### 2.13. Histological Analyses of Immunomodulatory and Osteogenesis Capability

**Figure 7:**
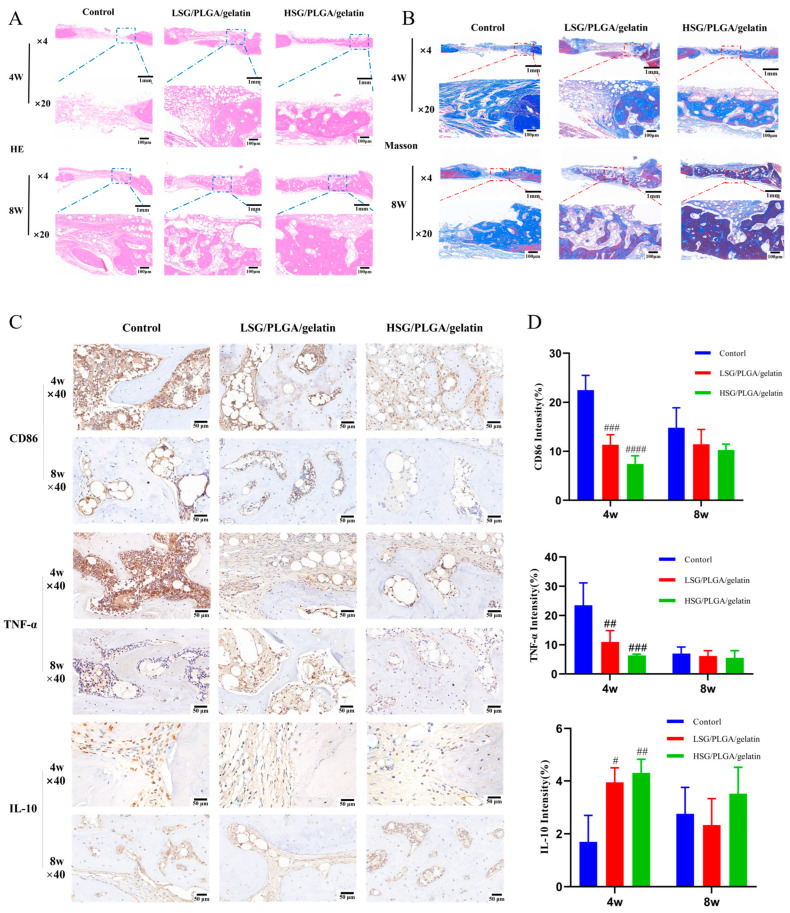
In vivo immunomodulatory and osteogenesis capability evaluations of SG. (**A**,**B**) HE and Masson staining, images obtained at 4× and 20×. (**C**,**D**) Immunohistochemical staining and quantification of CD86, TNF-α, and IL-10 (n = 3). Compared with the control group, # *p* < 0.05, ## *p* < 0.01, ### *p* < 0.001, #### *p* < 0.0001.

Hematoxylin and eosin (HE) and Masson staining revealed new bone formation across all groups at 4 and 8 weeks. As shown in Figure 7A,B, the control group exhibited bone defects filled with connective tissue and a sparse presence of osteoblasts. In contrast, the LSG and HSG groups demonstrated notable increases in osteoid tissue and newly formed bone. Specifically, in the HSG group, substantial amounts of osteoid tissue and newly formed woven bone were observed at 4 weeks, while near-complete bone regeneration, characterized by a mature bone matrix and trabeculae, was evident at 8 weeks.

CD86 and TNF-α, key markers of M1 macrophage polarization, and IL-10, a marker of M2 macrophages, were evaluated by immunohistochemistry. As shown in Figure 6C,D, CD86 and TNF-α expression were markedly elevated in the control group compared to the LSG and HSG groups at 4 weeks. In contrast, IL-10 levels were significantly higher in the HSG group than in the control and LSG groups at 4 weeks.

## 3. Discussion

Bone defects caused by diverse etiologies represent a major clinical burden due to their profound impact on patient quality of life. The interplay between bone regeneration and immune modulation, notably macrophage polarization into pro-inflammatory (M1) and anti-inflammatory (M2) phenotypes, is pivotal for effective recovery [23]. This study investigates the therapeutic potential of SG, a traditional Chinese medicinal herb, in modulating these immune pathways [24]. We employed a multifaceted approach, integrating network pharmacology and molecular docking analyses, to identify bioactive compounds within SG that interact with targets crucial for both bone healing and immune function. Our findings indicate that SG enhances osteogenic differentiation and promotes M2 macrophage polarization, thereby mitigating inflammatory responses. This dual action highlights the therapeutic potential of SG in addressing bone defects and integrating traditional medicine into contemporary regenerative frameworks.

The network pharmacology analysis shed light on the bioactive components of SG and their interactions with target genes crucial for bone regeneration and macrophage polarization. By utilizing the TCMSP Database, a starting point of 182 active ingredients was refined to 11 key compounds based on their DL and OB. This meticulous screening underscores the complexity of herbal formulations and emphasizes the need to identify multiple active compounds that enhance therapeutic efficacy. A total of 113 gene targets were associated with these active ingredients, highlighting the multi-targeted nature of SG. Notably, kaempferol and beta-sitosterol emerged as significant contenders due to their extensive interaction profiles, suggesting their vital roles in the therapeutic effects of SG. Kaempferol, a flavonoid aglycone, has been shown to inhibit interleukin-8 (IL-8) production while reducing levels of inflammatory cytokines such as IL-1β, interleukin-6 (IL-6), tumor necrosis factor-alpha (TNF-α), and transforming growth factor beta-1 (TGF-β1). This ability to modulate inflammatory pathways and alleviate oxidative stress is particularly noteworthy [25,26,27]. Similarly, beta-sitosterol displays anti-inflammatory activity by suppressing TNF-α and ROS secretion while enhancing antioxidant responses via superoxide dismutase (SOD) and glutathione peroxidase 4b (GPX4b) [28,29,30]. Interestingly, the analysis identified 33 crosstargets associated with both bone regeneration and macrophage polarization. Core targets, such as TNF and prostaglandin-endoperoxide synthase 2 (PTGS2), are implicated in inflammatory processes and macrophage activation. TNF-α orchestrates the immune response and drives the polarization of macrophages toward the M1 phenotype, which is essential for combating infections [31]. Exosomes derived from TNF-α-stimulated polymorphonuclear neutrophils significantly enhance M1 macrophage activation, elucidating a novel mechanism that could be targeted for therapeutic interventions [32]. Modulating these pathways towards an anti-inflammatory state, potentially supported by SG, is crucial for fostering an environment conducive to effective bone healing.

GO enrichment analysis revealed that the active components of SG significantly influence various biological processes, particularly in the positive regulation of apoptotic pathways and responses to xenobiotic stimuli. This indicates the potential role of SG in enhancing cell survival and proliferation while modulating crucial apoptotic mechanisms essential for effective tissue repair. Notably, SG exhibits a diverse range of pharmacological activities, including antioxidant, anti-inflammatory, and antidiabetic properties across different models. Its bioactive compounds, such as mogrosides and polysaccharides, act by modulating oxidative stress markers and reducing inflammation through the inhibition of pro-inflammatory cytokines like TNF-α and IL-6. Additionally, these compounds enhance glucose metabolism via the AMP-activated protein kinase (AMPK) pathway [33]. The regulation of immune responses and the enhancement of antioxidant enzyme activities highlight the capacity of SG to manage inflammation and metabolic disorders [34].

The KEGG pathway enrichment analysis further elucidated significant pathways associated with the identified targets, such as advanced glycation end-products and their receptor (AGE-RAGE) and TNF pathways. These pathways are instrumental in mediating inflammatory responses that may hinder bone regeneration. Interestingly, RAGE signaling, activated by glycated insulin, can impair insulin signaling, as demonstrated in CHO-IR-GLUT4 cells, where the phosphorylation of insulin receptors and AKT was compromised, leading to reduced glucose uptake [35]. Moreover, the inhibition of TNF-α signaling by fexofenadine and its subsequent effects on NF-κB activation in models of inflammatory arthritis highlight the importance of the TNF pathway [36]. In this context, piperazine ferulate protects against diabetic nephropathy by downregulating AGE-RAGE-mediated inflammatory signaling genes, illustrating the intricate connections between these pathways in inflammatory conditions [37]. Notably, *Bifidobacterium* has shown the ability to inhibit osteoclast formation while modulating the TNF-α/NF-κB pathway, suggesting a key role in the interplay between inflammation and bone regeneration [38].

PPI network analysis revealed complex interactions among identified targets, with core proteins exhibiting strong interconnectivity. This emphasizes that SG operates through a network of interactions rather than relying on a single target, embodying the systems biology approach inherent to TCM. These findings position SG as a promising herbal treatment for managing inflammation and promoting bone repair through multi-target mechanisms. For instance, mogrol, a key component of SG, inhibits osteoclastogenesis by downregulating various inflammatory markers and suppressing MAPK/NF-κB signaling, thereby reducing bone loss in postmenopausal mice [39]. Additionally, SG extract mitigates lipopolysaccharide (LPS)-induced intestinal inflammation by promoting M2 macrophage polarization and decreasing levels of pro-inflammatory cytokines [14]. These compelling results position SG as a valuable candidate for the treatment of inflammatory conditions such as bone defects.

Molecular docking established strong binding affinities between key targets like TNF, PTGS2, and caspase 3 (CASP3) with active components like kaempferol and beta-sitosterol. Subsequent MD simulations confirmed these interactions in a physiologically relevant environment. Four complexes, specifically kaempferol with TNF, PTGS2, JUN (jun proto-oncogene), and CASP3, underwent MD simulations to analyze stability over time. TNF–kaempferol and CASP3–kaempferol complexes exhibited remarkable stability, with RMSD values stabilizing after approximately 5 nanoseconds, suggesting that kaempferol consistently maintains orientation and interaction with protein targets, vital for therapeutic efficacy in bone repair. Fluctuation values were low, supporting effective binding without significant conformational changes leading to dissociation. The kaempferol–TNF complex demonstrated the highest hydrogen bond density, contributing to anti-inflammatory effects, particularly in the macrophage polarization context. Consistent Rg values suggest binding events do not significantly alter the overall shape and size of protein–ligand complexes, maintaining functional integrity. Structural stability in simulations suggests the potential for these compounds to exert effects in vivo, where conformational stability influences biological activity and efficacy.

In vitro studies demonstrate that SG effectively modulates macrophage behavior and enhances osteogenic differentiation within a co-culture system. Utilizing RAW 264.7 macrophages, an SG-conditioned medium was shown to modulate inflammatory responses and support bone repair mechanisms. Cytotoxicity assays revealed that SG extract concentrations of 200–800 µg/mL reduced macrophage viability, while 100 µg/mL was non-toxic, highlighting the necessity of dose optimization. Flow cytometry indicated that SG promotes macrophage polarization towards the M2 phenotype, as evidenced by increased CD206 and decreased CD86 expression, alongside reduced TNF-α and iNOS levels and elevated arginase 1 (Arg-1) and IL-10. This underscores SG’s potent anti-inflammatory effects, fostering an environment conducive to bone regeneration via enhanced differentiation of MC3T3-E1 osteoblast-like cells in SG-treated co-cultures. The involvement of paracrine signaling pathways is critical for osteoblast proliferation and differentiation, resonating with studies linking M2 macrophage polarization to osteogenesis through cytokine and growth factor release, such as BMPs and TNF. In vivo results confirm SG’s efficacy in immunomodulatory and bone regeneration. Histological analysis showed minimal bone formation in the control group, while the LSG and HSG groups exhibited increased osteoid tissue and new bone. The HSG group demonstrated substantial woven bone at 4 weeks and near-complete regeneration with mature trabeculae at 8 weeks, highlighting SG’s osteogenic potential. Immunohistochemical analysis revealed that SG significantly reduced the expression of CD86 and TNF-α while increasing IL-10 levels at 4 weeks. Given that CD86 and TNF-α are critical markers of M1 macrophages, their diminished expression in SG-treated groups indicates a shift away from pro-inflammatory activity. Conversely, the elevated IL-10, a marker of M2 macrophages, underscores SG’s capacity to promote an M2-dominant immune profile. This immunomodulatory effect likely contributes to the enhanced bone regeneration observed in the SG-treated groups, as M2 macrophages are known to secrete factors that stimulate osteoblast proliferation and differentiation. Supporting evidence from a diabetic rat model showed that an injectable hydrogel with mild hyperthermia accelerated bone formation by reducing ROS and inflammation and promoting M2 polarization [40]. Similarly, an immune-regulating hydrogel improved graft–bone integration in anterior cruciate ligament (ACL) reconstruction by fostering M2 polarization [41], while a biomimetic scaffold in steroid-induced osteonecrosis of the femoral head (SONFH) models diminished oxidative stress and enhanced bone repair [42]. These results underscore the potential of targeting immunomodulation for bone healing, suggesting SG as a promising therapeutic agent. Further in vivo studies and exploration of macrophage function and osteogenic signaling pathways are warranted, emphasizing the potential of SG in regenerative medicine and the therapeutic versatility of TCM herbal extracts.

However, limitations exist. While this study demonstrates the therapeutic potential of SG in bone repair both in vitro and in vivo, the clinical applicability of SG requires further exploration. Additionally, although key active compounds are identified, quantitative differences in concentrations or potential toxicity are not evaluated. Future research should rigorously assess the safety profiles and mechanisms of action of these components to optimize the therapeutic use of SG for bone regeneration in clinical settings. This comprehensive approach will enhance the reliability and effectiveness of SG in regenerative medicine.

## 4. Materials and Methods

### 4.1. Identification of Bioactive Components and Target Genes in SG

The chemical constituents of SG were analyzed using the TCMSP (https://tcmsp-e.com/, accessed on 5 October 2024) [43]. A preliminary screening process was conducted based on two key absorption, distribution, metabolism, and excretion (ADME) parameters: DL with a threshold of ≥ 0.18 and OB of ≥ 30%. Active compounds and their corresponding protein targets were identified through this screening.

Selected compounds were subsequently retrieved from the PubChem database (https://pubchem.ncbi.nlm.nih.gov/, accessed on 5 October 2024) [44] to obtain their Simplified Molecular Input Line Entry System (SMILES) representations. These representations were then submitted to the SwissADME database (www.swissadme.ch) [45] for further evaluation based on criteria including high gastrointestinal absorption and drug similarity, defined by two or more affirmative indicators. Following this screening, protein targets influenced by the compounds were standardized using the UniProt Protein Database (https://www.uniprot.org) [46] to ensure consistency in protein target information.

### 4.2. Identification of Target Genes in SG

Genes associated with bone defect formation were sourced from the GeneCards database (https://www.genecards.org/) [47]. Target genes of interest were selected based on a relevance score of ≥5 for both bone regeneration and macrophage polarization. The results were compiled and summarized, and duplicates were removed to create a comprehensive library of disease-related targets.

### 4.3. Construction of Protein–Protein Interaction (PPI) Network and Identification of Core Targets

Core targets were identified by overlapping SG potential targets with genes associated with bone defect formation. A Venn diagram was generated using Venny 2.1 (https://bioinfogp.cnb.csic.es/tools/venny/, accessed on 10 October 2024), and the intersecting targets were analyzed with the STRING database (https://string-db.org/) [48]. The analysis focused on Homo sapiens, applying a minimum interaction confidence threshold of greater than 0.4. Default settings were maintained for all other parameters to construct the PPI networks. The resulting networks were refined using Cytoscape version 3.10.2, and essential node centrality was evaluated using Cytoscape version 2.2 and Network Analyzer [49].

### 4.4. GO and KEGG Pathway Enrichment Analyses

Enrichment analyses for GO terms and KEGG pathways were conducted using the DAVID Knowledgebase v2024q2 functional annotation tool (https://davidbioinformatics.nih.gov/, accessed on 14 October 2024) [50]. Bar and bubble plots were created with the Bioinformatics platform (https://www.bioinformatics.com.cn). The mapping of target genes to KEGG pathways was performed using Cytoscape version 3.10.1.

### 4.5. Molecular Docking

Key targets and core compounds were selected from the protein–protein interaction and KEGG pathway network analyses. The Protein Data Bank (PDB) (https://www.rcsb.org) [51] provided the crystal structures of the key targets. Component structure MOL2 files were retrieved from the TCMSP database. The components were subjected to minimum free energy optimization using ChemBio3D and saved in MOL2 format. Protein receptors were prepared by removing solvent molecules and existing ligands using PyMOL, followed by optimization and saving in PDB format. AutoDockTools (version 1.57) was employed to convert the receptor and ligand files to PDBQT format and to define the active binding pocket. This process involved adding hydrogen atoms to the receptor, importing the ligand, setting the grid box spacing to 1.0, and exporting the configuration as GPF files. Molecular docking simulations were conducted using AutoDock Vina with an energy range of 5 and a maximum of 20 binding modes, executed via the command vina.exe --config config.txt --log log.txt --out output.pdbqt. The docking results, including minimum binding energies, were recorded. Binding diagrams were generated using PyMOL software. The output PDBQT files were integrated with the receptor files to generate PDB format complexes. These complexes were visualized with ligands highlighted in raspberry and receptors in light blue. Hydrogen bonds between ligands and receptors were identified and quantified, and the interacting amino acid residues were marked in light orange, with their names annotated.

### 4.6. MD Simulation

MD simulations were performed using GROMACS version 2020.3 (https://www.gromacs.org/). The CHARMM36 force field was applied to proteins, and the General Amber Force Field (GAFF) was utilized for ligands. The simulation box was configured to maintain a minimum distance of 1.0 nm between any protein atom and the box boundary and was solvated with water molecules at a density of 1. To ensure electrical neutrality, Cl⁻ and Na⁺ ions replaced appropriate water molecules. Energy minimization was executed using the steepest descent method for 5 × 10^4^ steps to eliminate unfavorable atomic contacts.

Equilibration proceeded in two phases: an NVT ensemble at 300 K for 100 ps to stabilize the temperature, followed by an NPT ensemble at 1 bar for 100 ps to stabilize the pressure. Production MD was conducted for 10 ns under isothermal–isobaric conditions of 300 K and 1 atm. Temperature and pressure were regulated using the V-rescale and Parrinello–Rahman thermostats with coupling constants of 0.1 ps and 0.5 ps, respectively. Van der Waals interactions were calculated using the Lennard–Jones potential with a cutoff distance of 1.4 nm. Bond lengths were constrained using the LINCS algorithm. Long-range electrostatic interactions were handled by the Particle Mesh Ewald method with a Fourier spacing of 0.16 nm.

### 4.7. Cell Culture

RAW 264.7 macrophages were cultured in a growth medium consisting of DMEM supplemented with 10% FBS and 1% penicillin–streptomycin (P/S) and were incubated at 37 °C in a humidified atmosphere containing 5% CO_2_. Cell densities varied based on the requirements of the experiments. Upon reaching the appropriate conditions, cells were prepared and seeded in 96-well, 24-well, and 6-well plates. Cells in optimal condition were selected for further study after undergoing the base treatment for the designated duration.

### 4.8. Preparation of SG-Conditioned Medium

To prepare the aqueous extract of SG (Beijing Solarbio Science & Technology Co., Ltd., Beijing, China), 10 g of SG was immersed in 100 mL of distilled water for 30 min, followed by decoction for 1 h. The resulting mixture was centrifuged at 10,000 rpm for 30 min to collect the supernatant. This extraction process was repeated twice, and the supernatants were combined and evaporated to dryness. The resulting dry powder was subsequently reconstituted in distilled water to achieve a concentration of 10 mg/mL. The solution was then filtered through a 0.22 μm pore-size filter and stored at −20 °C for future use.

### 4.9. Cell Viability and Proliferation

Proliferation of Raw 264.7 macrophages was assessed using the CCK-8 assay. Cells were seeded in 96-well plates at a density of 1.5 × 10^4^ cells per well and allowed to adhere for eight hours. The culture medium was then replaced with an SG-conditioned medium, and the cells were incubated for 48 h. Subsequently, 100 µL of SG-conditioned medium was added to each well. After washing with PBS, the cells were treated with a 10% CCK-8 solution in DMEM and incubated at 37 °C for one hour, ensuring that air bubbles were avoided during the process. Absorbance was measured at a wavelength of 450 nm.

### 4.10. Flow Cytometry Analysis

Flow cytometry was utilized to assess macrophage polarization. Raw 264.7 macrophages were co-cultured with SG for forty-eight hours before collection. The cells were then seeded in six-well plates at a density of 1 × 10⁶ cells per well and incubated with an SG-conditioned medium. Following this incubation, the cells were treated with 100 ng/mL lipopolysaccharide (LPS) and 20 ng/mL interleukins IL-4 and IL-13 for an additional forty-eight hours. After the treatment period, the cells were harvested and stained according to the manufacturer’s instructions. Flow cytometry analysis was performed to determine the expression levels of CD86 and CD206.

### 4.11. Quantitative Real-Time Polymerase Chain Reaction (qRT-PCR)

Total RNA was extracted from the treated cells and frozen ileum samples using TRIzol reagent (Takara), following the manufacturer’s instructions. The concentration and purity of the extracted RNA were assessed at 260 and 280 nm using an OD1000 spectrophotometer. The reaction system and procedures were established following the SYBR Green Detection Kit (Takara) guidelines. Data analysis utilized the 2^−ΔΔCt^ method, with GAPDH serving as the reference control. The primers for RT-qPCR were synthesized by TaKaRa Biotechnology Co., Ltd. (Dalian, China) and are listed in Table 4.

### 4.12. Osteogenic Differentiation Assay

A Transwell co-culture system was employed to evaluate the effect of SG-conditioned macrophages on the osteogenic differentiation of MC3T3-E1 cells (Figure 6H). Raw 264.7 macrophages were seeded in Transwell inserts with 0.4 µm pores at a density of 1 × 10⁵ cells per well in six-well plates. After a 48 h exposure to SG-conditioned medium, the medium was removed, and the cells were rinsed with PBS. Subsequently, MC3T3-E1 cells were plated in flat-bottom six-well Transwell plates at a density of 1 × 10^6^ cells per well and co-cultured with the SG-treated macrophages in an osteogenic induction medium. Alkaline phosphatase (ALP) staining was performed on day seven to assess osteogenic differentiation.

### 4.13. Preparation of SG/PLGA/Gelatin Composite Scaffolds

10% w/v PLGA solution was prepared by dissolving a measured amount of PLGA in dichloromethane. NaCl particles of varying sizes were sieved and incorporated into the PLGA solution at a PLGA-to-NaCl ratio of 1:15. The mixture was thoroughly stirred until homogeneous and cast into circular molds with a diameter of 5 mm and a thickness of 0.5 mm. The molds were placed in a fume hood overnight to ensure complete evaporation of dichloromethane. After demolding, two single-layer PLGA modules with distinct pore sizes were obtained. Dichloromethane was utilized to bond the single-layer modules together. The bonded constructs were immersed in deionized water for three days, with the solvent replaced every eight hours to facilitate the complete dissolution of the NaCl porogen. The modules were subsequently freeze-dried for 24 h.

PLGA modules were then soak-loaded with freshly prepared phosphate-buffered saline (PBS) as the control and 25 µg/mL (LSG) and 100 µg/mL (HSG) SG-conditioned media. Gelatin (Sigma-Aldrich, St. Louis, MO, USA) was accurately weighed and dissolved in distilled water at 50 °C using a water bath. The gelatin solution was diluted with triple-distilled water to achieve a final concentration of 12% and stirred in a 37 °C water bath for two hours to ensure complete dissolution. The SG/PLGA microspheres were immersed in the prepared gelatin solution and subjected to ultrasonic vibration for 30 min to promote thorough infiltration of gelatin into the microsphere pores. After cooling, the gelatin was allowed to solidify, and the samples were dehydrated by immersion in anhydrous ethanol for four hours. Finally, the dehydrated samples were cross-linked with chlorine dioxide for twelve hours at 25 °C. This comprehensive process resulted in the successful preparation of the SG/PLGA/gelatin composite bone implant material.

### 4.14. Calvarial Defect Model

All animal experiments received approval from the Ethics Committee of Guangxi Medical University. Six-week-old rats weighing between 150 and 180 g were utilized for in vivo bone regeneration assessments. Ten rats were randomly assigned to three groups, with the blank group serving as the control. Anesthesia was induced using sodium pentobarbital. Following the shaving and disinfection of the surgical site, an incision was made to expose the cranial bone. Using an orthopedic drill, two 5 mm defects were created on either side of the cranial suture, and PBS-, LSG-, or HSG-loaded scaffolds were implanted into the defects. After implantation, the incisions were closed in layers. Animals were housed under a 12 h light/dark cycle with ad libitum access to a standard laboratory diet. At 4 weeks and 8 weeks post-implantation, the rats were euthanized, and the samples were collected and fixed in 4% paraformaldehyde for subsequent analysis.

### 4.15. Histological Evaluation

Samples were decalcified in 10% EDTA for 60 days, with the solution being replaced every 2 days. Following decalcification, the samples were embedded in paraffin, and serial sections of 3 μm thickness were prepared using a pathology slicer. Hematoxylin and eosin (HE) staining was conducted to assess new bone formation. Additionally, immunohistochemical staining for TNF-α was performed to evaluate macrophage polarization and function. After deparaffinization, rehydration, and antigen retrieval, the sections were incubated overnight at 4 °C with primary antibodies against CD86 (1:200, Abmart, Shanghai, China), TNF-α (1:500, Proteintech, Wuhan, China), and IL-10 (1:500, Servicebio, Wuhan, China). Following washing, the sections were incubated with secondary antibodies (1:1000, Solarbio, BeiJing, China) for 1 h, with three samples analyzed per group. Confocal microscopy was employed for observation and imaging, and ImageJ 1.53e software was utilized for statistical analysis.

### 4.16. Statistical Analysis

All quantitative data in this study are expressed as the mean ± standard deviation (SD), with each experiment conducted in triplicate. Statistical significance was assessed using one-way ANOVA with Tukey’s multiple comparisons test via GraphPad Prism 8.4.2 software. Statistically significant differences are indicated by # *p* < 0.05, ## *p* < 0.01, and ### *p* < 0.001 as significant differences versus the blank control group. * *p* < 0.05, ** *p* < 0.01, and *** *p* < 0.001 indicate significant differences versus the positive control group.

## 5. Conclusions

In this study, an integrative strategy combining network pharmacology and experimental validation revealed the therapeutic potential of SG in bone defect repair. Mechanistically, SG drives macrophage polarization toward the M2 phenotype, reduces inflammatory responses, and enhances osteogenic differentiation. Notably, molecular docking analysis identified kaempferol and beta-sitosterol as key bioactive compounds exhibiting high-affinity binding to critical regulatory targets, including TNF, PTGS2, and CASP3, directly linking their roles to the therapeutic efficacy of SG. These findings align with our network pharmacology results, which uncovered 33 overlapping targets associated with bone regeneration and macrophage reprogramming, collectively supporting the utility of SG in accelerating bone repair and advancing regenerative medicine strategies.

## Figures and Tables

**Table 1:** Bioactive compounds of SG.

Mol ID	Molecule Name	OB(%)	DL
MOL009295	flazin	94.27	0.38
MOL002140	perlolyrine	65.94	0.27
MOL010105	(S)-2-methylbutyl-4-(4-decyloxybenzylideneamino)	45.01	0.7
MOL001749	ZINC03860434	43.59	0.34
MOL001494	mandenol	41.99	0.19
MOL000422	kaempferol	41.88	0.24
MOL010131	mogroester	41.68	0.31
MOL010070	11-oxomogroside IIA1	37.62	0.22
MOL000358	beta-sitosterol	36.91	0.75
MOL001506	supraene	33.54	0.42
MOL010072	11-oxomogroside IIE	32.77	0.21

**Table 2:** Topological characterizing of core targets.

Gene Names	Uniprot ID	Protein Names	Degree	Betweenness	Closeness
*TNF*	P01375	Tumor necrosis factor	30	104.0552585	0.941176
*PTGS2*	P35354	Prostaglandin G/H synthase 2	28	64.36478232	0.888889
*PPARG*	P37231	Peroxisome proliferator-activated receptor gamma	27	58.5240787	0.864865
*CASP3*	P42574	Caspase-3	25	25.35190353	0.820513
*JUN*	P05412	Transcription factor Jun	25	30.21752402	0.820513
*HSP90AA1*	P07900	Heat shock protein HSP 90-alpha	19	28.56351398	0.711111
*BCL2*	P10415	Apoptosis regulator Bcl-2	25	23.21023686	0.820513
*RELA*	Q04206	Transcription factor p65	24	57.11209028	0.800000
*TGFB1*	P01137	Transforming growth factor beta-1 proprotein	24	19.89555432	0.800000
*ICAM1*	P05362	Intercellular adhesion molecule 1	22	30.17604244	0.761905
*STAT1*	P42224	Signal transducer and activator of transcription 1-alpha/beta	21	15.2762432	0.744186
*HMOX1*	P09601	Heme oxygenase 1	21	9.067893137	0.744186
*VCAM1*	P19320	Vascular cell adhesion protein 1	19	16.00899837	0.711111
*IKBKB*	O14920	Inhibitor of nuclear factor kappa-B kinase subunit beta	19	7.896653347	0.711111
*MAPK8*	P45983	Mitogen-activated protein kinase 8	18	8.979000629	0.695652

**Table 3:** Docking results between component and hub genes.

Component	Targets	Vina Score	Component	Targets	Vina Score
kaempferol	TNF	−8.60	beta-sitosterol	JUN	−7.50
kaempferol	PTGS2	−9.20	beta-sitosterol	HSP90AA1	−6.60
kaempferol	PPARG	−8.40	beta-sitosterol	CASP3	−7.30
kaempferol	JUN	−7.40	flazin	PTGS2	−9.60
kaempferol	HSP90AA1	−7.20	flazin	HSP90AA1	−8.20
kaempferol	CASP3	−7.30	perlolyrine	PTGS2	−9.20
beta-sitosterol	PTGS2	−9.20	mandenol	PTGS2	−6.00

**Table 4:** Primer sequence.

Gene	Primer Sequence
*iNOS*	5′-ATGGCTCGGGATGTGGCTAC-3′ (Forward)
	3′-ACTTCTATAGAAGCCACGTCAGAAA-5′ (Reverse)
*TNF-α*	5′-GCCAGGAGGGAGAACAGAAACTC-3′ (Forward)
	3′-ACAGGGAAAGTGAGTGACCGG-5′ (Reverse)
*Arg-1*	5′-AGCTCTGGGAATCTGCATGG-3′ (Forward)
	3′-ATAGACGGTTTCTGTAGCACATGTA-5′ (Reverse)
*IL-10*	5′-TAGAGCTGCGGACTGCCTTC-3′ (Forward)
	3′-CTTCGTACCGGGTCTTTAGT-5′ (Reverse)
*GAPDH*	5′-TGTGTCCGTCGTGGATCTGA-3′ (Forward)
	3′-GAGGACGCTGAAGTTGTCGTT-5′ (Reverse)

## Data Availability

Data will be available on request.

## Abbreviations

SG	*Siraitia grosvenorii*
TCM	Traditional Chinese Medicine
TNF	tumor necrosis factor
IL	interleukin
iNOS	Inducible Nitric Oxide Synthase
ARG	arginase
MAPK	mitogen-activated protein kinase
NF-κB	nuclear factor-kappa B
PPI	protein–protein interaction
GO	Gene Ontology
KEGG	Kyoto Encyclopedia of Genes and Genomes
MD	molecular dynamics
ROS	Reactive Oxygen Species
OB	oral bioavailability
DL	drug-likeness
RMSD	root mean square deviation
FCM	flow cytometry
ALP	alkaline phosphatase
